# Nanoreactor-Structured Defective MoS_2_: Suppressing Intercalation-Induced Phase Transitions and Enhancing Reversibility for Potassium-Ion Batteries

**DOI:** 10.1007/s40820-025-01992-x

**Published:** 2026-01-05

**Authors:** Chunrong Ma, Cyrus Koroni, Jiacheng Hu, Ji Qian, Guangshuai Han, Hui Xiong

**Affiliations:** 1 School of Mechanical and Electrical Engineering, Qingdao University, Qingdao, 266071 People’s Republic of China; 2 Micron School of Materials Science and Engineering, Boise State University, Boise, ID 83725 USA; 3 Shandong Key Laboratory of Advanced Chemical Energy Storage and Intelligent Safety, Advanced Technology Research Institute, Beijing Institute of Technology, Jinan, 250300 People’s Republic of China; 4 School of Automotive Studies, Tongji University, Shanghai, 201804 People’s Republic of China

**Keywords:** Potassium ion batteries, Phase transitions, Structure reversibility, Intercalated heterostructure, Defect engineering

## Abstract

Conversion-type electrode materials hold significant promise for potassium-ion batteries (PIBs) due to their high theoretical capacities, yet their practical deployment is hindered by sluggish kinetics and irreversible structural degradation. To overcome these limitations, we propose a rationally engineered nanoreactor architecture that stabilizes defect-rich MoS_2_ via interlayer incorporation of a carbon monolayer, followed by encapsulation within a nitrogen-doped carbon shell, forming a MoSSe@NC heterostructure. This tailored structure synergistically accelerates both K^+^ diffusion kinetics and electron transfer, enabling unprecedented rate performance (107 mAh g^−1^ at 10 A g^−1^) and ultralong cyclability (86.5% capacity retention after 1200 cycles at 3 A g^−1^). Mechanistic insights reveal a distinctive “adsorption-conversion” pathway, where sulfur vacancies on exposed S–Mo–S basal planes act as preferential K^+^ adsorption sites, effectively suppressing parasitic phase transitions during intercalation. In situ X-ray diffraction and transmission electron microscopy corroborate the structural reversibility of the conversion reaction, with the carbon matrix dynamically accommodating strain while preserving electrode integrity. This work not only advances the understanding of defect-driven interfacial chemistry in conversion-type materials but also provides a versatile strategy for designing high-performance anodes in next-generation PIBs through heterostructure engineering.

**Supplementary Information:**

The online version contains supplementary material available at 10.1007/s40820-025-01992-x.

## Highlights

- A nanoreactor-structured MoSSe@NC heterostructure was constructed via defect engineering and carbon intercalation, simultaneously achieving phase transition suppression and enhanced ion transport.
- Selenium-induced lattice disorder and carbon layer confinement synergistically inhibit the 1T–2H phase transition and buffer structural strain during cycling.
- The designed heterostructure exhibits high capacity, excellent rate performance, and long-term cycling stability, offering a generalizable strategy for high-performance potassium-ion battery anodes.

**Supplementary Information:**

The online version contains supplementary material available at 10.1007/s40820-025-01992-x.

## Graphical abstract

**Figure:**
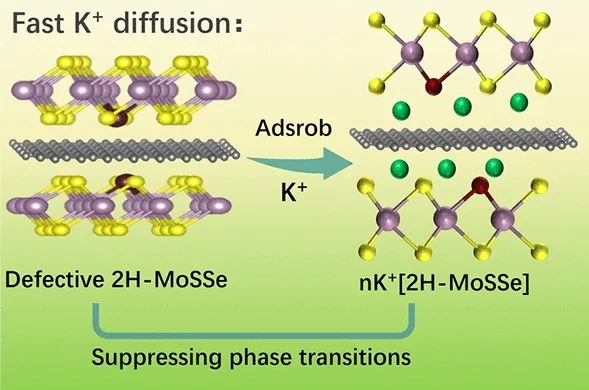

## Introduction

**Fig. 1:**
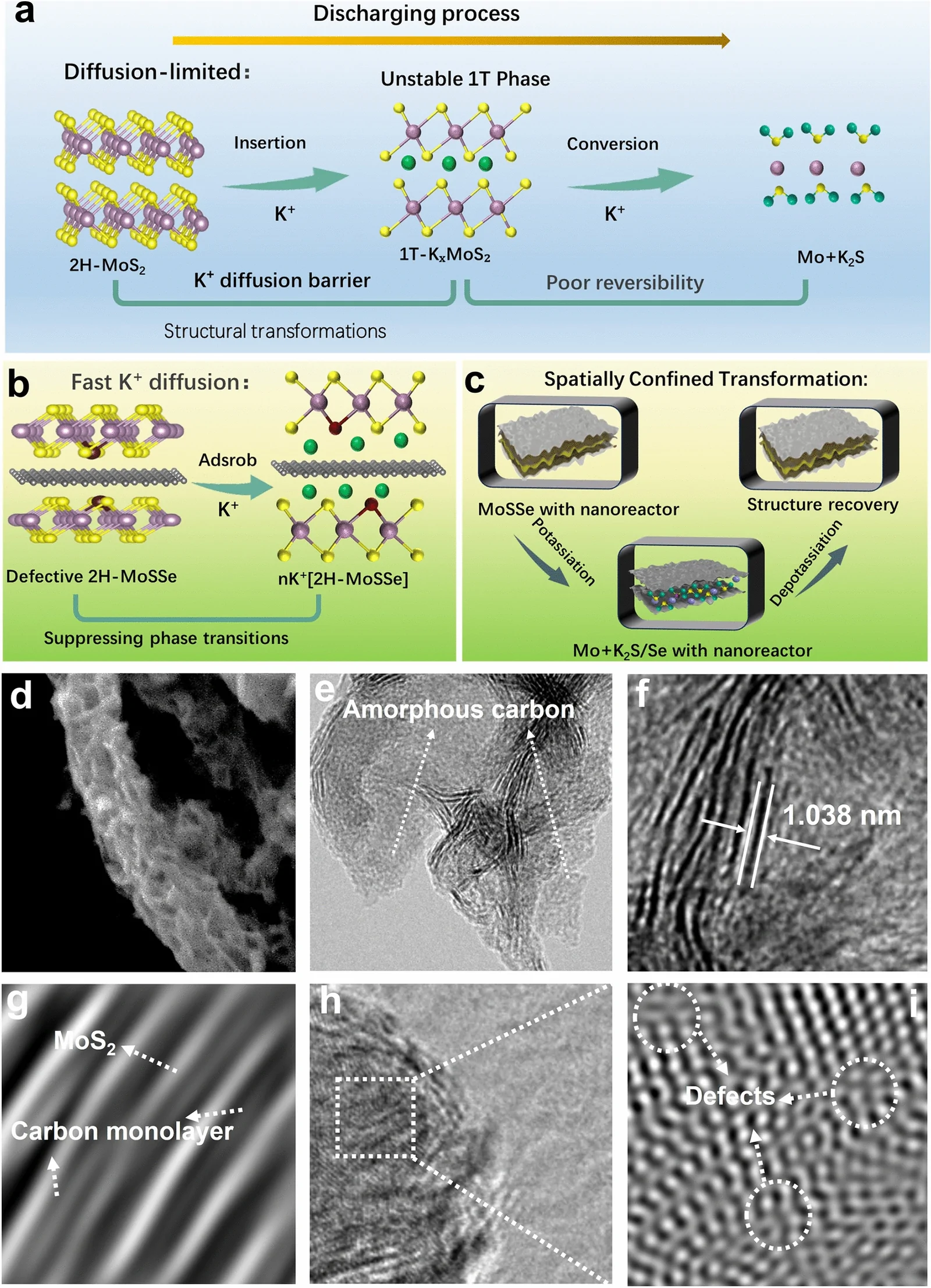
**a** Schematic illustration of phase evolution in MoS_2_ anode during discharge. **b** MoSSe@NC suppresses phase transition. **c** Spatially confined transformation in MoSSe@NC. **d** SEM image of MoSSe@NC. **e**, **f** TEM images. **g-i** HRTEM images highlighting the structural features of MoSSe@NC

Molybdenum disulfide (MoS_2_), a layered transition metal dichalcogenide, has attracted considerable attention as a promising anode material for potassium-ion batteries (PIBs) due to its high theoretical capacity (~ 670 mAh g^−1^) and tunable interlayer spacing [1–4]. The van der Waals gaps between the S–Mo–S layers create natural pathways for K^+^ intercalation, promoting the electrochemical reaction. In addition, MoS_2_ exhibits a conversion reaction mechanism that enables multi-electron transfer, which is essential for improving capacity. However, the practical application of MoS_2_ in PIBs is hindered by several key challenges. One of the main issues is the first-order phase transition that occurs during K^+^ intercalation, wherein MoS_2_ transitions from the K-deficient 2H phase to the K-rich 1 T phase [5, 6]. This phase transition introduces significant kinetic barriers, as ion diffusion is coupled with phase boundary migration, thereby limiting the rate performance of the material (Fig. 1a). As a result, capacity retention of MoS_2_ at current densities above 2 A g^−1^ typically drops below 50%, restricting its application in high-power scenarios. Another challenge arises during deep discharge (< 1.0 V), when the conversion reaction produces metallic Mo and K_2_S. The irreversible formation of K_2_S during discharge leads to persistent structural defects that prevent reversible conversion upon charging [7]. Incomplete reconversion of K_2_S exacerbates capacity loss, while residual K_2_S promotes oxidative dissolution at higher voltages (> 2.0 V), resulting in the formation of soluble polysulfides. This polysulfide dissolution induces a shuttle effect similar to that in the potassium–sulfur system, which accelerates degradation and results in rapid Coulombic efficiency decay (Fig. 1a). Consequently, addressing the interrelated issues of sluggish kinetics, irreversible phase transitions, and interfacial instability has become essential.

To overcome the limitations related to phase transitions and structural instability, recent studies have increasingly emphasized the structural optimization of MoS_2_, aiming to enhance its electrochemical performance and improve long-term cycling stability. A key strategy involves the controlled expansion of interlayer spacing [8–11], which reduces ion diffusion barriers and facilitates the intercalation of potassium ions. Additionally, precise regulation of material morphology at the nanoscale [12–15], such as the formation of few-layered or vertically aligned structures, can effectively shorten ion transport pathways and increase the density of electrochemically active sites. To further enhance performance, MoS_2_ is often composited with conductive carbon-based frameworks [16–19], which not only improve electronic conductivity but also provide structural support that alleviates mechanical stress during repeated cycling. In parallel, electrolyte engineering has emerged as an effective strategy to regulate electrode–electrolyte interfaces and stabilize interphases for potassium-ion storage [20]. For example, constructing a K_2_SO_3_-rich interphase through tailored electrolyte design has been demonstrated to significantly improve cycling stability and rate capability. Furthermore, the adsorption behavior of potassium ions is closely governed by the electronic configuration of the host lattice, particularly the degree of electron delocalization. In this context, defect engineering has emerged as a promising approach to tailor the electronic environment and enhance electrochemical activity [21–24]. Among various strategies, anion doping and atomic substitution have proven effective in introducing structural defects that facilitate charge redistribution, improve electronic conductivity, and promote potassium-ion storage kinetics. Regulation of interlayer spacing and Mo–S bonding through heteroatom incorporation further contributes to the stabilization of the K^+^ intercalation process and reduction of diffusion barriers. However, despite these advances, heteroatom-doped or substituted systems often suffer from complex phase transitions, leading to disintegration or rearrangement of the original crystal structure, which poses a significant challenge to structural stability. Notably, the influence of doping on the reconversion kinetics has received limited attention, highlighting the necessity for further investigation into its role in enhancing material reversibility. Furthermore, to date, no studies have reported on conversion-type materials in 2D-layered systems that can withstand substantial phase and volume changes while maintaining performance throughout charge/discharge cycles, particularly for materials undergoing deep conversion reactions in PIBs.

In this study, we propose a unique nanoreactor design to stabilize defect-engineered MoS_2_ by intercalating a carbon monolayer between the layers of MoSSe, thereby constructing a heterostructure that is subsequently encapsulated with nitrogen-doped carbon (MoSSe@NC). This dual-engineered structure not only enhances mechanical and structural integrity but also introduces multiple functionalities critical for potassium-ion storage. Specifically, selenium incorporation induces abundant defects and lattice disorder, which effectively suppress phase transitions typically triggered by potassium-ion intercalation, and more importantly, significantly accelerate ion transport kinetics (Fig. 1b). Moreover, the inserted carbon monolayer functions as a structurally stable interlayer, expanding the interlayer distance to alleviate electrostatic repulsion that impedes ion migration (Fig. 1c). Meanwhile, the system creates a confined, atomic thin two-dimensional space that facilitates the structural transformation of MoSSe with enhanced reversibility. Simultaneously, the nitrogen-doped carbon shell acts as an efficient nanoreactor, accommodating volume changes during cycling and suppressing the dissolution and shuttle of polysulfide species [25–27]. This contributes to a stable reaction environment and significantly improves long-term cycling performance. The synergistic effect of defect engineering and nanoreactor confinement is clearly supported by in situ X-ray diffraction (in situ XRD) and transmission electron microscopy (in situ TEM) analyses, which reveal the effective mitigation of structural degradation and suppression of undesired phase evolution during electrochemical cycling. As a result, the MoSSe@NC composite exhibits exceptional potassium storage performance, delivering a high-rate capability of 125 mAh g^−1^ at 10 A g^−1^ with an ultrafast 15-s charge/discharge time, and outstanding cycling stability, retaining 90% of its capacity after 1200 cycles at 3 A g^−1^.

## Experimental Section

### Materials

Sodium molybdate dihydrate (Na_2_MoO_4_·2H_2_O, analytical grade), ammonium molybdate ((NH_4_)_6_Mo_7_O_24_·4H_2_O), thiourea (CH_4_N_2_S), glucose, dopamine hydrochloride, selenium (Se) powder, and ammonia solution (NH_3_·H_2_O, 25–28%) were all obtained from Sinopharm Chemical Reagent Co., Ltd. Multi-walled carbon nanotubes (CNTs, > 95%) were purchased from Chengdu Organic Chemicals Co., Ltd., Chinese Academy of Sciences. All chemical reagents were used as received without further purification. Deionized water and analytical grade ethanol were employed throughout all experimental procedures.

### Materials Synthesis

The preparation of the CNT–MoS_2_ precursor involved a two-stage process. Initially, a hydrothermal reaction was conducted. Specifically, 0.2 g of sodium molybdate dihydrate (Na_2_MoO_4_·2H_2_O), 0.4 g of thiourea (CH_4_N_2_S), 0.05 g of glucose, and 30 mg of acid-treated multi-walled carbon nanotubes (CNTs) were dispersed in deionized water through ultrasonic treatment for 30 min to achieve uniform mixing. The obtained homogeneous suspension was transferred into a Teflon-lined stainless steel autoclave and maintained at 200 °C for 24 h. After cooling naturally to room temperature, the resulting products were repeatedly rinsed with deionized water and ethanol to remove unreacted residues and byproducts. In the subsequent step, 0.4 g of the previously obtained CNT–MoS_2_ was dispersed in a mixed solvent of 80 mL ethanol and 20 mL water. Then, 0.3 g of ammonium molybdate and 0.1 g of dopamine hydrochloride were introduced into the suspension, followed by magnetic stirring for 5 min. The pH of the mixture was adjusted to approximately 10 by dropwise addition of ammonia solution, and the reaction was allowed to proceed at ambient temperature for 10 h. The resulting products were collected by centrifugation, thoroughly washed with water and ethanol, and subsequently dried. The dried composite powder was then blended with selenium (Se) powder and placed in a quartz boat within a tubular furnace. The system was heated under an argon atmosphere to 700 °C at a ramping rate of 5 °C min^−1^ to complete the selenization process. For reference, a MoS_2_@NC sample was prepared following the same protocol but without the introduction of Se powder. In addition, pristine MoS_2_ was synthesized using an identical hydrothermal approach, except that glucose was excluded from the precursor solution.

### Materials Characterizations

The morphology and microstructure of the obtained samples were examined using field emission scanning electron microscopy (FE-SEM, JEOL JSM-7800F) and transmission electron microscopy (TEM, JEOL JEM-2100Plus). The crystalline phase information was determined by X-ray diffraction (XRD, Rigaku SmartLab) equipped with Cu Kα radiation (*λ* = 1.5406 Å). Raman spectra were collected on a Jobin Yvon T6400 spectrometer to characterize the carbon-related structural features. The chemical composition and valence states of the elements were analyzed by X-ray photoelectron spectroscopy (XPS, PHI 5000 VersaProbe III).

### Electrochemical Measurements

The electrochemical performance was investigated using CR2032-type coin cells assembled in an argon-filled glove box. Potassium metal served as both the counter and reference electrode. The working electrode was prepared by mixing the active material, Super P, and sodium carboxymethyl cellulose (CMC) binder in a weight ratio of 8:1:1 to form a homogeneous slurry, which was then uniformly coated on copper foil and dried under vacuum at 90 °C for 10 h. A glass fiber membrane (Whatman GF/D) was employed as the separator, and the electrolyte consisted of 1 M KPF_6_ dissolved in a 1:1 (v/v) mixture of ethylene carbonate (EC) and diethyl carbonate (DEC) with 5 vol% fluoroethylene carbonate (FEC) additive. Galvanostatic charge–discharge measurements were carried out on a LAND-CT3002A testing system within a potential window of 0.01–3.0 V (vs. K^+^/K). Cyclic voltammetry (CV) was performed at various scan rates using a CHI650E electrochemical workstation. The mass loading of the active material on each electrode was approximately 1.2 mg cm^−2^. The specific capacity was calculated based on the active material mass, and the Coulombic efficiency was determined as the ratio of the charge capacity to the discharge capacity.

## Results and Discussion

### Structural Design and Characterization

As shown in Fig. 1a, 2H-MoS_2_ undergoes sequential structural transformations during potassiation, including potassium-ion insertion, formation of a metastable 1 T phase, and subsequent conversion to metallic Mo and K_2_S. The large ionic radius of K^+^ and the relatively narrow van der Waals gap in 2H-MoS_2_ contribute to a high diffusion barrier, which is frequently accompanied by pronounced lattice distortion and poor phase reversibility. These structural instabilities often lead to rapid capacity fading and unsatisfactory long-term cycling behavior. To address these limitations, a dual-engineering strategy was implemented, as illustrated in Fig. 1b. Selenium doping into the MoS_2_ lattice introduces abundant anion vacancies and structural defects, enhancing surface reactivity and providing additional K^+^ adsorption sites. The defective MoSSe framework facilitates faster ion diffusion and helps suppress phase transitions commonly observed in pristine MoS_2_. Moreover, the insertion of carbon monolayers between the MoSSe layers leads to an expanded interlayer spacing, which reduces electrostatic repulsion and lowers the energy barrier for K^+^ transport. The resulting two-dimensional confinement environment not only enhances ion accessibility but also contributes to the structural stabilization of the layered host during cycling. The morphology of the synthesized MoSSe@NC was analyzed using scanning electron microscopy (SEM) and transmission electron microscopy (TEM). As shown in Fig. 1d, the MoSSe nanosheets are uniformly dispersed on the carbon nanotube (CNT) surface without noticeable aggregation. The TEM image (Fig. 1e) reveals that the MoSSe nanosheets are fully encapsulated by a two-dimensional (2D) amorphous carbon layer. High-resolution TEM (HRTEM, Fig. 1f) images indicate the presence of approximately 3–4 layers with an interlayer spacing of 1.0 nm, in contrast to the annealed MoS_2_ nanosheets, which exhibit a well-ordered layered structure with an interlayer spacing of 0.62 nm (Fig. S1). Furthermore, the HRTEM analysis shows slight twisting of the (002) planes, attributed to the intercalation of a carbon monolayer into the MoS_2_ lattice [28, 29]. A magnified image in Fig. 1g distinctly highlights the uniform incorporation of a carbon monolayer between the interlayers of MoSSe, forming a well-defined intercalated architecture. Detailed TEM observations (Fig. 1h, i) further reveal the presence of characteristic defects within the MoSSe nanosheets, including lattice cracks and sulfur vacancies, which are indicative of the defect-engineered nature of the structure. In contrast, the MoS_2_@NC sample (Fig. S2) exhibits a relatively intact morphology without pronounced structural defects, suggesting the absence of deliberate defect modulation in the MoS_2_@NC composite. Elemental mapping images confirm the uniform distribution of Mo, S, Se, N, and C across the defective MoSSe@NC structure (Fig. S3).

**Fig. 2:**
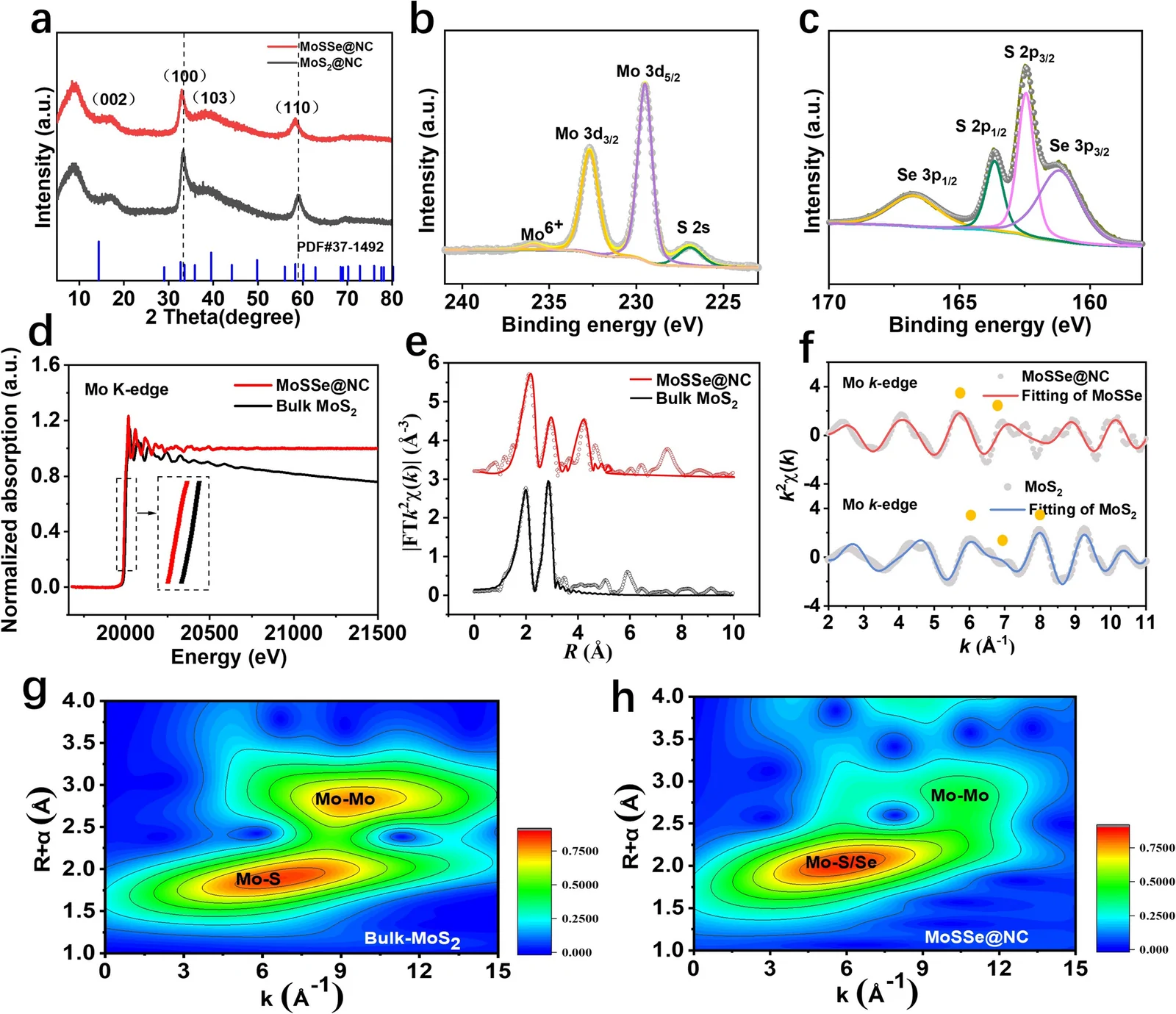
**a** XRD patterns of MoS_2_, MoSSe@NC, and MoS_2_@NC. **b** Mo 3*d* XPS spectrum of MoSSe@NC. **c** S 2*p* XPS spectrum of MoSSe@NC. **d** Normalized XANES spectra at the Mo K-edge for MoSSe@NC and MoS_2_. **e** *k*^2^-weighted Fourier-transformed EXAFS spectra of MoSSe@NC and MoS_2_. **f** EXAFS fitting curves in *k*-space (*k*^2^-weighted) for MoSSe@NC and MoS_2_. **g**, **h** WT contour plots of EXAFS for MoS_2_ and MoSSe@NC

The XRD patterns of MoSSe@NC, presented in Fig. 2a, exhibit well-defined diffraction reflections that can be indexed to the 2H-MoS_2_ phase (JCPDS PDF#89–5112). Notably, in comparison with pristine MoS_2_, the characteristic (002) reflection at 13.8° is split into two distinct diffraction features centered at 8.8° and 16.8°. This splitting behavior is consistent with previously reported characteristics of single-layer MoS_2_ nanosheets [30], indicating that the restacking of MoS_2_ layers is effectively inhibited in the MoSSe@NC hybrid. The observed interlayer spacing of approximately 1.0 nm in the MoSSe@NC sample aligns well with the predicted structural model, further confirming the expanded layered architecture. In addition, a noticeable shift of the diffraction signals toward lower angles is observed for MoSSe@NC compared to MoS_2_@NC, suggesting an enlarged interlayer distance as a result of Se incorporation [31]. The shift can be attributed to the substitution of sulfur with selenium (Se), which effectively expands the interlayer distance and facilitates ion diffusion. Furthermore, X-ray photoelectron spectroscopy (XPS) was conducted to analyze the surface chemical state of as-prepared MoSSe@NC (Fig. S4). The high-resolution C 1*s* and N 1*s* spectra (Fig. S5a, b) of MoSSe@NC reveal distinct peaks associated with C–C and C–N/C=N bonds for carbon species [32], as well as graphitic-N, pyrrolic-N, and pyridinic-N for nitrogen species [33]. Additionally, the spectra indicate the formation of Mo–C and Mo–N bonds, confirming the interaction between Mo atoms and the C/N atoms in the N-doped carbon monolayers. The high-resolution Mo 3*d* spectrum (Fig. 2b) of MoSSe@NC displays two prominent peaks at 232.3 and 229.1 eV, corresponding to Mo^4+^, with a slight shift to lower binding energies compared to MoS_2_@C [21]. Similarly, the S 2*s* peaks also shift to lower binding energies, appearing at 232.3 and 229.1 eV, likely due to the incorporation of selenium (Se). As shown in Fig. 2c, the S 2*p* peaks at 161.5 eV (2*p*_3/2_) and 162.8 eV (2*p*_1/2_) correspond to S^2−^ in Mo–S bonds. Additionally, Se 3*p* peaks at 163.2 eV (3*p*_3/2_) and 164.5 eV (3*p*_1/2_) indicate the presence of Se^2−^ in Mo–Se bonds, confirming the incorporation of Se into the lattice [34]. Raman spectroscopy was performed to analyze the defect characteristics of the carbon materials in the MoSSe@NC system (Fig. S6). For the carbon nanotubes, the intensity ratio of the D to G band (I_D_/I_G_) is 0.89, indicating a relatively low defect density and a partially graphitized structure. After introducing the intercalated carbon, the I_D_/I_G_ ratio increases to 1.20, suggesting the generation of more structural defects. As shown in Fig. S7, the thermal stability and weight loss behavior of the MoSSe@NC composite were evaluated by thermogravimetric analysis (TGA). As the temperature increased from room temperature to approximately 430 °C, the MoSSe@NC composite exhibited an initial weight loss of 9.7%, which is primarily attributed to the decomposition of the embedded carbonaceous components. Upon further heating to 800 °C, a significant weight reduction was observed, mainly due to the combustion of CNTs. The total carbon content in the MoSSe@NC composite is estimated to be approximately 21.8%. The surface properties were evaluated by Brunauer–Emmett–Teller (BET) analysis (Fig. S8). The MoSSe@NC exhibits a type IV adsorption–desorption isotherm, indicating a mesoporous structure, with a measured specific surface area of 158.3 m^2^ g^−1^. X-ray absorption spectroscopy (XAS) was employed to further investigate the atomic and electronic structures. The Mo K-edge of the MoSSe@NC composite (Fig. 2d) shifts to a lower energy compared to pristine MoS_2_, indicating significant electronic structure modulation due to Se doping [35]. Extended X-ray absorption fine structure (EXAFS) analysis (Fig. 2e) at the Mo K-edge reveals three main peaks at approximately 1.9, 2.8, and 4.3 Å, corresponding to Mo–S/Se, Mo–Mo, and Mo–C/N coordinate covalent bonds, respectively, whereas only Mo–S and Mo–Mo bonds are detected in pristine MoS_2_. Notably, the first shell in MoSSe@NC exhibits a red shift compared to MoS_2_, confirming the formation of Mo–Se bonds due to Se doping [36]. Additionally, the MoSSe@NC composite shows reduced Mo–Mo coordination intensity compared to MoS_2_, suggesting a decreased Mo–Mo coordination number, attributed to the formation of Mo–C and Mo–N bonds during intercalation [37]. Distinct oscillation pattern in the 6–8 k Å^−1^ range, which differs from that observed in the spectrum of pristine MoS_2_. The Mo K-edge EXAFS signals provide insights into the local atomic environment around the central Mo atoms. The unique oscillatory feature observed in MoSSe@NC indicates a change in the local structure around the central Mo atoms, which can be attributed to the incorporation of Se atoms [23]. This suggests that Se doping induces a modification in the Mo–S bonding, with Se atoms likely coordinating to the Mo centers through Mo–Se. The EXAFS wavelet transform (WT) analysis (Fig. 2g, h) of Mo K-edge EXAFS oscillations reveals that the WT maximum for the Mo–Mo bond in MoSSe@NC shifts from ∼9.8 Å^−1^ in MoS_2_ to ∼10.9 Å^−1^. This shift suggests a reduced population of Mo–Mo bonds in MoSSe@NC, attributed to the coordination of Mo atoms with lighter elements such as C and N.

### Electrochemical Measurements and Analysis

**Fig. 3:**
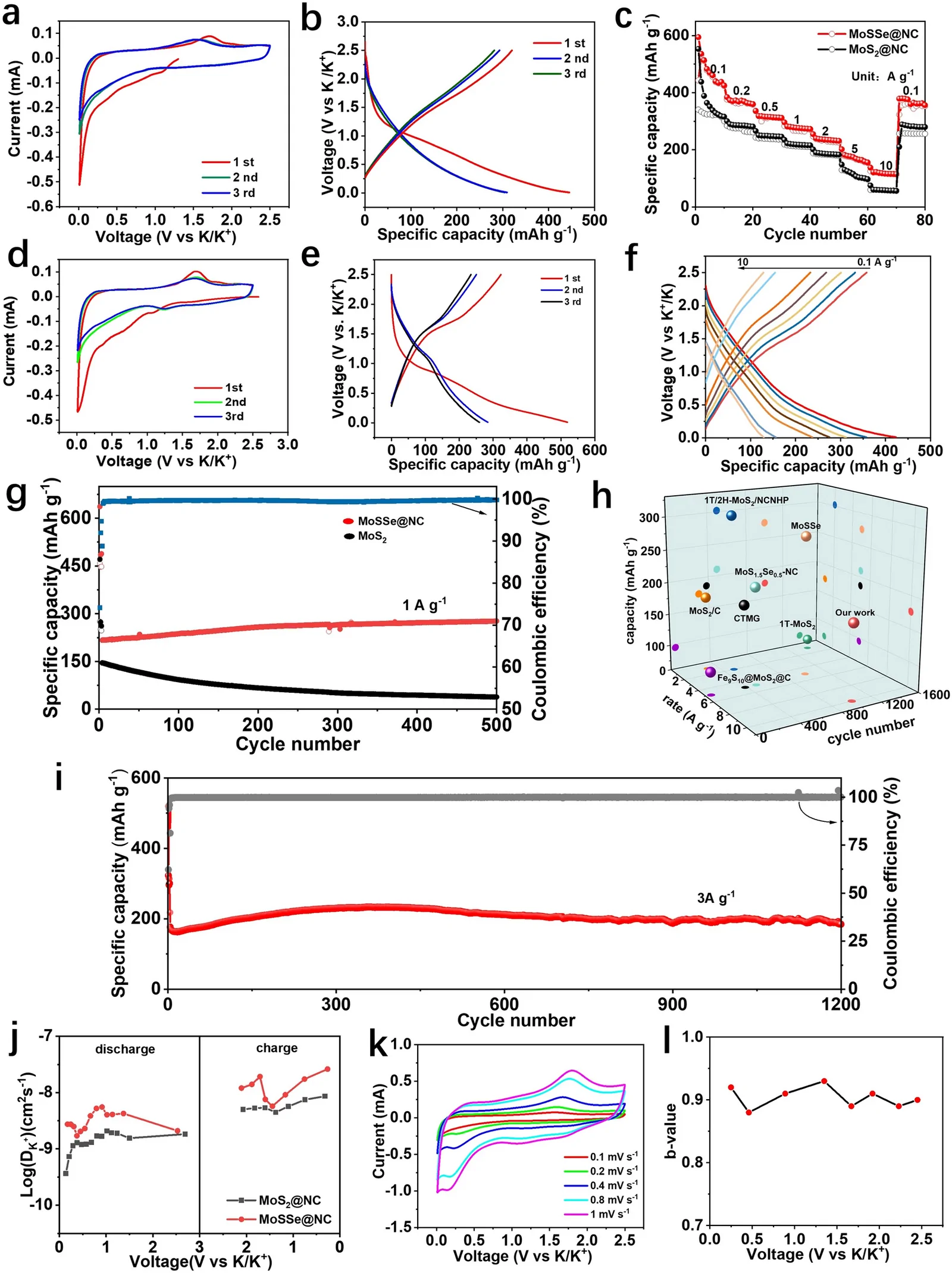
**a**, **d** CV curves of the MoSSe@NC and MoS_2_@NC electrode at 0.1 mV s^−1^. **b** Galvanostatic charge–discharge profiles of the MoSSe@NC electrode at 1 A g^−1^. **c** Rate capabilities of MoSSe@NC and MoS_2_@NC electrodes. **e** Charge–discharge profiles of the MoS_2_@NC electrode at 1 A g^−1^. **f** Charge–discharge profiles the MoSSe@NC electrode at different rate. **g** Cycling performance of MoSSe@NC and MoS_2_ electrodes at 1 A g^−1^. **h** Comparison of the electrochemical performance of the MoSSe@NC electrode with literature data. **i** Long-term cycling performance of the MoSSe@NC electrode at 3 A g^−1^. **j** D_K+_ coefficient in MoSSe@NC and MoS_2_@NC electrodes. **k** CV curves of the MoSSe@NC electrode at various scan rates; **l** Corresponding b-values

The potassium-ion storage performance of the MoSSe@NC composite was evaluated using potassium-ion half-cells. The cyclic voltammetry (CV) curves obtained at a scan rate of 0.1 mV s^−1^ (Fig. 3a) exhibit a distinct cathodic peak at approximately 1.1 V in the initial cycle, which is absent in subsequent scans. This peak corresponds to the irreversible formation of the solid electrolyte interphase (SEI) layer [38, 39]. In addition, a cathodic peak at 0.26 V is observed, attributed to the conversion reaction of MoS_2_ to metallic Mo and K_2_S, following the reaction: MoS_2_ + 4 K^+^ + 4e^−^ → 2 K_2_S + Mo [40, 41]. Starting from the second cycle, only the 0.26 V peak remains, indicating that the conversion reaction becomes the dominant and reversible process. In contrast, a peak around 1.2 V, commonly associated with potassium-ion intercalation into the MoS_2_ lattice and typically observed in MoS_2_@NC composites (Fig. 3d) and other MoS_2_-based electrodes [41–44], is not detected in the MoSSe@NC composite. The absence of this peak implies that the intercalation process is significantly suppressed. Instead, potassium ions are primarily stored via surface adsorption on the few-layered MoS_2_ nanocrystals through an interfacial storage mechanism, which is indicative of capacitive-controlled charge storage behavior. The predominance of capacitive contribution facilitates rapid charge transfer and structural stability during cycling, thereby enhancing the rate capability and long-term durability of the MoSSe@NC electrode. The anodic peak centered at approximately 1.7 V is assigned to the reversible conversion of metallic Mo back to the MoSSe phase [11]. The near-complete overlap of the CV curves in subsequent cycles further confirms the high reversibility of the redox reactions and indicates structural stability of the MoSSe@NC electrode during repeated potassiation/depotassiation processes. The galvanostatic charge–discharge profiles further validate the potassium storage behavior of the MoSSe@NC electrode, as shown in Fig. 3b. When tested between 0.01 and 2.5 V at a current density of 1 A g^−1^, the first three discharge–charge curves exhibit sloping profiles without distinct voltage plateaus, suggesting a predominantly capacitive-dominated storage mechanism. In the initial cycle, MoSSe@NC delivers a discharge capacity of 448.6 mAh g^−1^ and a charge capacity of 323.5 mAh g^−1^, corresponding to an initial Coulombic efficiency (ICE) of 72% (Table S1). The irreversible capacity loss is mainly attributed to the formation of the SEI and electrolyte decomposition during the first potassiation process. In the subsequent second, the discharge capacities stabilize at 318.0 and 310.6 mAh g^−1^, with a significantly improved coulombic efficiency of 97%, indicating enhanced electrochemical reversibility after initial activation. By contrast, the MoS_2_@NC electrode (Fig. 3e) exhibits a distinct discharge plateau, indicative of a typical intercalation-conversion process. The first discharge and charge capacities are 513.0 and 308.0 mAh g^−1^, respectively, resulting in a lower ICE of 60%. The MoSSe@NC composite exhibits superior rate performance compared to the MoS_2_@NC electrode, as shown by the rate capability data in Fig. 3c. Specifically, the MoSSe@NC electrode maintains stable specific capacities of 312, 285, 268, and 207 mAh g^−1^ at current densities of 0.1, 0.5, 2.0, and 5.0 A g^−1^, respectively. Remarkably, even at an ultrahigh current density of 10.0 A g^−1^, the electrode retains 52.3% of its initial capacity, demonstrating its exceptional kinetic stability. Moreover, after 10 cycles of high-rate testing, when the current density is reduced back to 0.1 A g^−1^, the electrode recovers 95.6% of its original capacity, further highlighting its outstanding reversibility and structural integrity. The galvanostatic charge/discharge profiles of MoSSe@NC at various current densities exhibit sloping curves without pronounced plateaus, suggesting a predominantly capacitive-dominated potassium-ion storage mechanism (Fig. 3f). As the current density increases from 0.1 to 10 A g^−1^, the overall curve shape remains well preserved, indicating favorable rate performance and fast reaction kinetics. To evaluate the structural stability and long-term cycling performance, the electrochemical behavior of MoSSe@NC and pristine MoS_2_ was compared at a current density of 1 A g^−1^ over 500 cycles (Fig. 3g). The MoSSe@NC composite exhibited a steady capacity increase during initial cycles, eventually stabilizing at approximately 290 mAh g^−1^, with a high Coulombic efficiency consistently maintained around 99%–100%. In contrast, the pristine MoS_2_ electrode showed a rapid capacity decay, dropping below 100 mAh g^−1^ within the first 100 cycles and continuing to deteriorate thereafter. The structural reversibility of MoSSe@NC was further examined by selected-area electron diffraction (SAED) after prolonged cycling. As shown in Fig. S9, distinct diffraction rings corresponding to the (100) and (110) planes can be clearly identified. Importantly, these characteristic rings are consistently observed both before and after 500 charge–discharge cycles, indicating that the layered structure is well preserved and the crystallographic framework remains stable during long-term operation. The MoSSe@NC electrode exhibits exceptional long-term cycling stability under high-rate conditions, as shown in Fig. 3i. After 1200 cycles at a high current density of 3 A g^−1^, the MoSSe@NC electrode retains a reversible capacity of 180 mAh g^−1^, which corresponds to a capacity retention of 86.5% compared to its initial value. Notably, the capacity curves show minimal fluctuations throughout the cycling process, with a decay rate as low as 0.011% per cycle, indicating the mechanical stability and electrochemical reversibility of MoSSe@NC electrode. Compared with other MoS_2_-based anodes (Fig. 3h; see details and references in Table S2), the MoSSe@NC electrode exhibits significantly enhanced electrochemical performance, including superior rate capability, higher specific capacity, and exceptional long-term stability. The K-ion full cell was assembled using MoSSe@NC as the anode and K_3_V_2_ (PO_4_)_3_ as the cathode, as illustrated in Fig. S10. The charge/discharge voltage profiles at a current density of 0.5 A g^−1^ within a voltage window of 0.01–3 V are shown in Fig. S10a. The full cell delivers an initial charge/discharge capacity of 261/176 mAh g^−1^, corresponding to an ICE of 67%. As presented in Fig. S10b, the full cell exhibits stable cycling performance at 0.5 A g^−1^. After several activation cycles, the CE stabilizes at approximately 98%. Ultimately, the full cell delivers a reversible capacity of around 110 mAh g^−1^, demonstrating its promising potential for practical potassium-ion storage applications. The potassium-ion diffusion kinetics of the electrodes were quantitatively assessed using the galvanostatic intermittent titration technique (GITT), with the corresponding diffusion coefficients (D_k_^+^) calculated based on Fick’s second law (detailed derivation provided in Fig. S11) [45]. During both discharge and charge processes, MoSSe@NC exhibits markedly higher D_k_^+^ values (10^−8^–10^−9^ cm^2^ s^−1^) than MoS_2_@NC (10^−9^–10^−1^⁰ cm^2^ s^−1^), demonstrating accelerated K^+^ transport kinetics. The elevated D_k_^+^ of MoSSe@NC in the discharge process further evidences that Se substitution effectively promotes ionic diffusion. This enhancement arises from the enlarged interlayer spacing and defect-rich structure introduced by Se incorporation, which facilitate ion migration. Consequently, the superior rate capability of MoSSe@NC can be ascribed to its optimized ion diffusion characteristics, underscoring the beneficial role of heteroatomic modification in improving potassium-ion storage kinetics. As shown in the Nyquist plots (Fig. S12), the MoSSe@NC electrode exhibits a much smaller semicircle compared with MoS_2_@NC, indicating a substantially reduced charge-transfer resistance. This result demonstrates that Se doping effectively promotes charge transport and accelerates the electrochemical reaction kinetics. The improved reaction dynamics can be ascribed to the generation of anion vacancies and structural defects induced by Se incorporation, which not only enhance surface reactivity but also create additional K^+^ adsorption sites, thereby facilitating more efficient electrochemical processes. To elucidate the electrochemical kinetics of the MoSSe@NC electrode, CV measurements were carried out at scan rates ranging from 0.2 to 1.0 mV s^−1^ (Fig. 3k). The CV curves at different scan rates maintain similar profiles with clearly distinguishable redox peaks, and only slight shifts in peak positions are observed with increasing scan rate, indicating favorable reaction reversibility and fast potassium-ion transport kinetics. The dependence of peak current (i) on scan rate (v) follows the power-law relationship i = av^b^ [46], where the fitted b-values for both cathodic and anodic peaks exceed 0.8, suggesting that the charge storage process is primarily governed by surface-induced capacitive behavior (Fig. 3l). To further quantify the contribution of capacitive and diffusion-controlled processes, the current response was analyzed according to the equation i(v) = k_1_v + k_2_v^1/2^, where k_1_v corresponds to the capacitive component and k_2_v^1/2^ represents the diffusion-controlled contribution [46]. As illustrated in Fig. S13, the capacitive contribution accounts for up to 80% of the total current at a scan rate of 0.2 mV s^−1^. This enhanced pseudocapacitive behavior can be attributed to the unique heterostructure of MoSSe@NC, where defect engineering, expanded interlayer spacing, and the conductive carbon matrix collectively facilitate rapid ion transport and surface reactions.

### Mechanistic Analysis of Potassium Storage

**Fig. 4:**
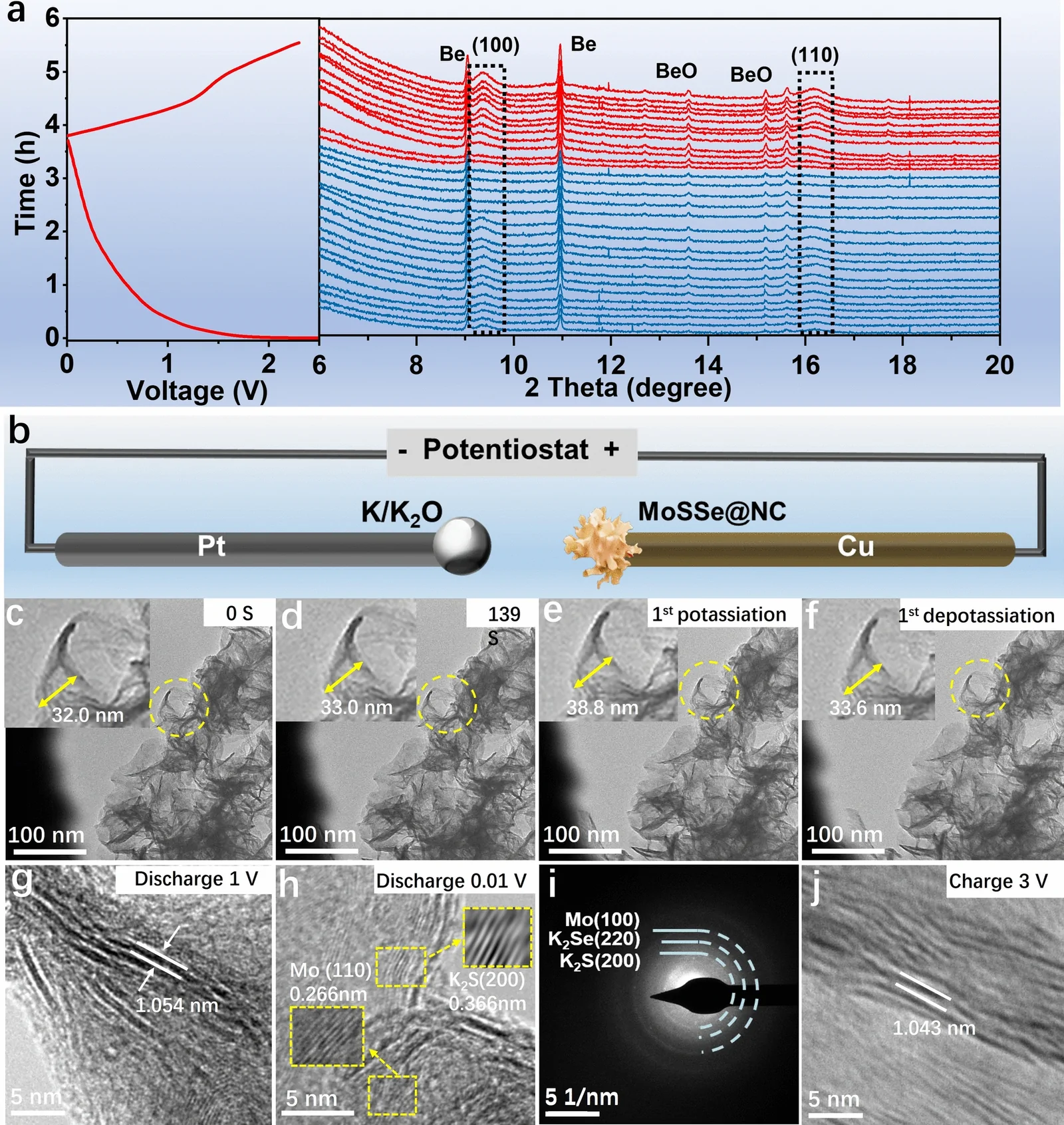
**a** In situ XRD patterns of the MoSSe@NC electrode and the corresponding charge–discharge profile. **b** Schematic illustration of the in situ TEM experimental setup. **c**–**f** In situ TEM images captured at different electrochemical states during the charge–discharge process. **g** Ex situ TEM image of the electrode discharged to 1.0 V. **h** Ex situ TEM image of the electrode discharged to 0.01 V. **i** Corresponding selected SAED pattern of the fully discharged state. **j** Ex situ TEM image of the electrode after being recharged to 3.0 V

A series of in situ/ex situ characterizations were conducted to monitor the phase and structural evolution of the MoSSe@NC electrode during the charge/discharge process. Figure 4a shows the in situ XRD patterns of MoSSe@NC during a complete charge/discharge cycle. During the discharge process (3.0 → 0.01 V), two distinct peaks at approximately 9.5° and 11.8°, corresponding to the (100) and (110) planes of MoSSe@NC, are observed in the initial XRD pattern. These peaks remain stable until the voltage reaches 0.3 V, where K^+^ ions are adsorbed onto the surface active sites, forming a [K^+^]_n_[MoSSe] compound. As discharge continues, the (100) and (110) peaks gradually weaken and disappear when the voltage reaches 0.01 V, accompanied by the typical conversion reaction of MoSSe (MoSSe → K_2_S/K_2_Se). Notably, no K^+^-intercalation compounds are detected, indicating that the first phase transition in the MoSSe@NC electrode is suppressed. During the subsequent charge process (0.01 → 2.5 V), the peaks gradually reappear at the same positions and become stronger as the voltage increases to 2.5 V. These results suggest that the intercalated heterostructure of MoSSe@NC is largely recovered from the disordered amorphous Mo + K_2_S/K_2_Se after a complete cycle. In situ TEM observations were conducted to elucidate the K^+^ storage mechanism and track the microstructural evolution of MoSSe@NC during potassiation and depotassiation. The experimental setup for the PIBs in a half-cell configuration is illustrated in Fig. 4b. A potential of − 5 V was applied in the nanobattery configuration to initiate potassiation (refer to Video S1), resulting in a minor volume expansion. As shown in Fig. 4d, after 139 s of potassiation, the length of the MoSSe nanosheet increased slightly from approximately 32 nm (Fig. 4c) to 33 nm. After the first potassiation (Fig. 4e), the length of the MoSSe nanosheets increased slightly by approximately 1.5% compared to the original size. Notably, MoSSe preserved its original morphology throughout the entire potassiation process, showing no signs of structural damage or significant deformation. Moreover, after complete depotassiation (Fig. 4f), the length of the MoSSe nanosheets nearly returned to its original size. The phase transformation of MoSSe@NC was further examined using ex situ TEM images at different stages (Fig. 4g). After discharging to 1 V, the spacing between the MoSSe layers remained almost unchanged, as Na^+^ ions adsorb onto the surface without intercalating into the structure. As the discharge proceeded to 0.01 V (Fig. 4h), the layered MoSSe structure nearly vanished, and the Mo and K_2_S phases emerged. The corresponding selected-area electron diffraction (SAED) pattern (Fig. 4i) clearly detected the Mo and K_2_S/K_2_Se phases. Upon full charging to 3 V (Fig. 4j), the Mo and K_2_S/K_2_Se products from discharge underwent reversible conversion, leading to the reformation of the layered MoSSe structure with an interlayer spacing of 1.0 nm, which closely matched its original value. Based on the above in situ and ex situ characterizations, a plausible “adsorb-conversion” mechanism is proposed to explain the reversible reconstructability of MoSSe@NC. The MoSSe nanosheets, intercalated with carbon monolayers, are confined within the nanoreactor. Upon discharging to 1 V, the typical insertion reaction does not occur; instead, K^+^ ions adsorb onto the surface, while the MoSSe layers and the intercalated heterostructure remain well preserved. Further discharging to 0.01 V induces the typical conversion reaction, accompanied by the destruction of MoSSe layers, during which MoSSe is converted to Mo and K_2_S/K_2_Se. Upon charging to 3 V, the Mo and S/Se atoms recombine, resulting in the formation of a 2D-layered MoSSe structure between the carbon monolayers.

**Fig. 5:**
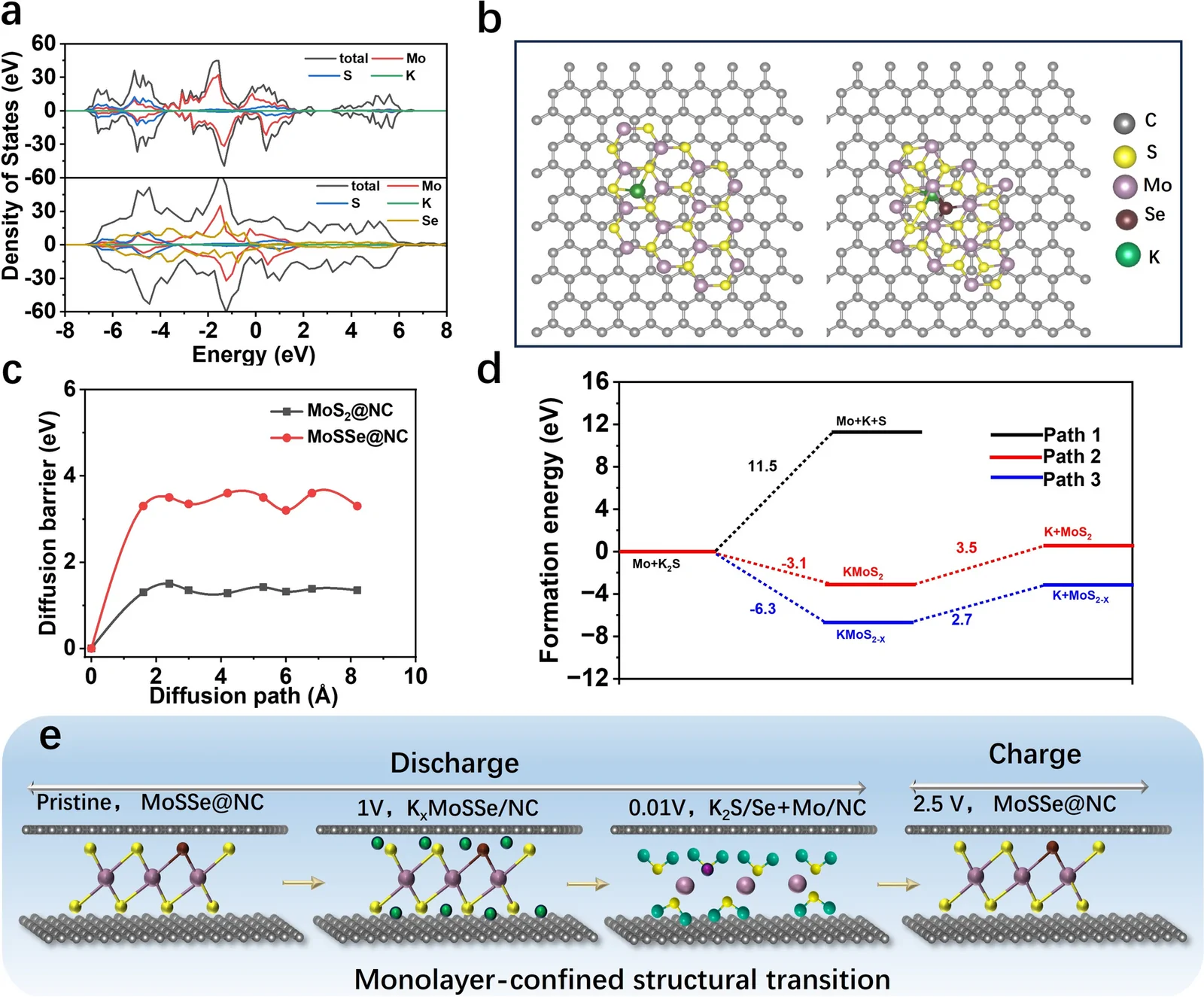
**a** Calculated DOS of MoSSe@NC and MoS_2_@NC. **b** Binding energies of K^+^ intercalation sites in MoSSe@NC and MoS_2_@NC. **c** Diffusion energy barriers of K^+^ in MoSSe@NC and MoS_2_@NC. **d** Formation energies of MoSSe@NC and MoS_2_@NC based on first-principles calculations. **e** Schematic illustration of the monolayer-confined structural transition

To better understand the reversible conversion of MoS_2_ enhanced by the bifunctional nanoreactor, density functional theory (DFT) calculations were carried out. As depicted in Fig. S14, a single-layer MoS_2_/N-doped graphene structure with a single Se atom was constructed to model MoSSe@NC. For comparison, a similar structure without Se was also constructed to represent MoS_2_@NC. The electronic conductivity and K^+^ diffusion energy barriers were calculated to investigate the reaction kinetics during discharge. As shown in Fig. 5a, both the total and partial density of states (DOS) indicate that MoSSe@NC exhibits significantly improved electrical conductivity relative to MoS_2_@NC. Additionally, the calculated binding energy for K^+^ intercalation in MoSSe@NC is − 1.96 eV (Fig. 5b), which is more negative than that for MoS_2_@NC (− 1.47 eV), suggesting that MoSSe@NC has a stronger affinity for K^+^ adsorption. The K^+^ diffusion paths in both materials are shown in Figs. 5c and S15. The K^+^ diffusion energy barrier in the MoSSe@NC is 0.78 eV, which is significantly lower than the barrier in the MoS_2_/NC (3.19 eV). This demonstrates that the introduction of selenium atoms into MoS_2_ facilitates faster K^+^ diffusion. To investigate the reversibility of the reactions, the reaction pathways and their respective formation energies for the reverse processes involving Mo and K_2_S during charging were calculated. As depicted in Fig. 5d, the formation energy for the reaction pathway resulting in Mo + K + S is significantly higher (9.26 eV) than that for the formation of KMoS_2_, suggesting that the latter pathway is energetically more favorable. Furthermore, the introduction of defects, such as Se substitution for sulfur, significantly reduces the formation energies of both KMo_1-x_S_2_ and MoS_2_, suggesting that such defects are essential for enhancing the kinetics of the conversion reactions. Based on the preceding analysis, a plausible mechanism for the reversible reconstruction of the MoSSe@NC intercalated heterostructure is proposed, which involves a “monolayer-confined structural transition”. As shown in Fig. 5e, the intercalation of carbon monolayers within the MoSSe layers creates a layered structure consisting of S/Se-Mo–S and carbon, with MoSSe monolayers confined between two parallel carbon monolayers. Upon discharging to 1 V, K^+^ ions adsorb at the MoSSe@NC interface (K_x_MoS_2_@NC), during which the MoSSe layers and the intercalated heterostructure are largely maintained. As the voltage is further reduced to 0.01 V, a typical conversion reaction takes place, leading to the disruption of MoS_2_ layers and the transformation of MoSSe into Mo and K_2_S/K_2_Se within the carbon interlayer. Notably, despite the breaking of Mo–S bonds during this phase transition, the relative positions of Mo and S atoms are largely preserved due to the confinement imposed by the carbon monolayers and the stabilizing Mo–C/N interactions. Upon charging to 2.5 V, Mo and S/Se atoms undergo recombination to form a two-dimensional layered MoSSe structure confined between carbon monolayers. This reconstruction is driven by the templating and spatial confinement effects imposed by the carbon layers, thereby enabling the reversible formation of the intercalated MoSSe@NC heterostructure.

## Conclusions

In summary, a well-defined nanoreactor architecture has been constructed by inserting a carbon monolayer between MoS_2_ layers, introducing selenium through partial sulfur substitution to form MoSSe, and coating the entire structure with a nitrogen-doped carbon shell, enabling highly reversible conversion reactions in potassium-ion batteries. The deliberate introduction of sulfur vacancies within the MoS_2_ lattice provides abundant electron-rich sites that promote preferential K^+^ adsorption, thereby initiating a distinctive “adsorption–conversion” mechanism and effectively suppressing irreversible phase transitions typically associated with ion intercalation. The embedded carbon monolayer not only enhances the electronic conductivity but also guides the reversible reconstruction of MoS_2_ domains, further contributing to structural stability during cycling. Selenium doping plays a dual role by expanding the interlayer spacing to 0.79 nm, which facilitates rapid K^+^ transport, and concurrently reducing charge transfer resistance, thus accelerating the overall electrochemical kinetics. As a result of these coordinated structural and compositional modifications, the MoSSe@NC electrode delivers a high-rate capacity of 160 mAh g^−1^ at 10 A g^−1^ and retains 86.5% of its capacity after 1200 cycles, clearly outperforming conventional MoS_2_-based anodes. These findings not only deepen the understanding of defect-induced conversion mechanisms in layered transition metal dichalcogenides but also provide a practical design strategy for developing high-performance anode materials for next-generation energy storage systems.

## Supplementary Information

Below is the link to the electronic supplementary material.

Supplementary file1 (DOCX 2318 KB)

Supplementary file2 (MP4 3791 KB)
