# Supplementary material for: Nanoreactor-Structured Defective MoS2: Suppressing Intercalation-Induced Phase Transitions and Enhancing Reversibility for Potassium-Ion Batteries
Source: Nanomicro Lett. 2026 Jan 5;18:138. doi: 10.1007/s40820-025-01992-x (PMC12765779; doi:10.1007/s40820-025-01992-x)
Supplement: Supplementary file 1 — Supplementary file1 (DOCX 2318 KB) [file 40820_2025_1992_MOESM1_ESM.docx]

Supporting Information for

**Nanoreactor-Structured Defective MoS_2_: Suppressing Intercalation-Induced Phase Transitions and Enhancing Reversibility for Potassium-Ion Batteries**

Chunrong Ma^1^, Cyrus Koroni^2^, Jiacheng Hu^2^, Ji Qian^3^, Guangshuai Han^4*^, and Hui Xiong^2*^

^1^ School of Mechanical and Electrical Engineering, Qingdao University, Qingdao 266071, P. R. China

^2^ Micron School of Materials Science and Engineering, Boise State University, Boise, ID 83725, United States

^3^ Shandong Key Laboratory of Advanced Chemical Energy Storage and Intelligent Safety, Advanced Technology Research Institute, Beijing Institute of Technology, Jinan 250300, P. R. China

^4^ School of Automotive Studies, Tongji University, Shanghai 201804, P. R. China

*Corresponding authors. E-mail: [17666026@tongji.edu.cn](mailto:17666026@tongji.edu.cn) (Guangshuai Han); [clairexiong@boisestate.edu](mailto:clairexiong@boisestate.edu) (Hui Xiong)

**Supplementary Figures and Tables**


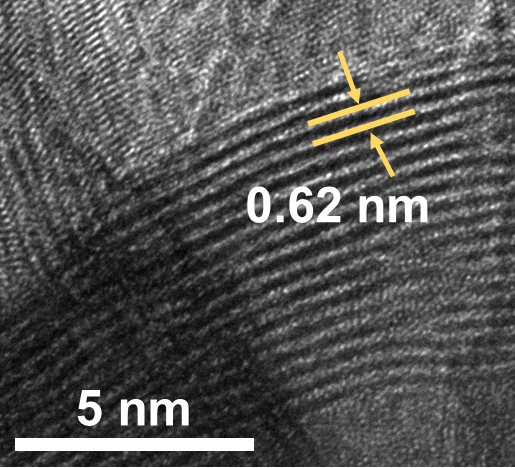


**Fig. S1** TEM image of pure MoS_2_


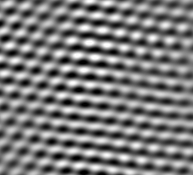


**Fig. S2** HRTEM image of MoS_2_@NC


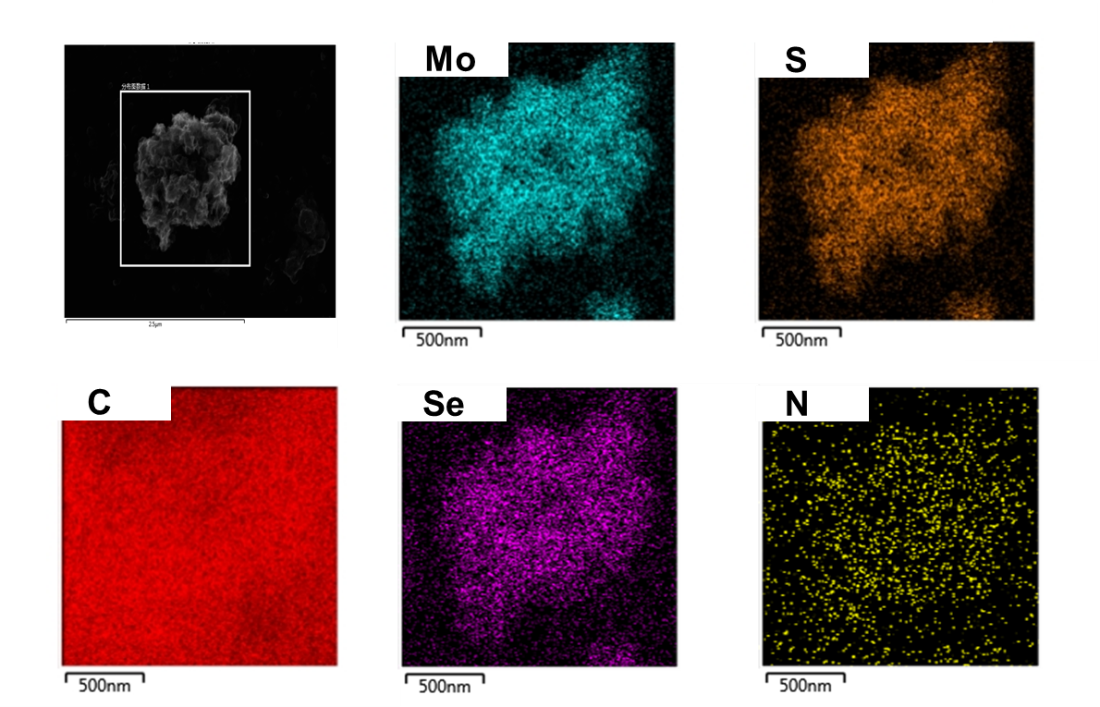


**Fig. S3** Elemental mapping images of MoSSe@NC composite


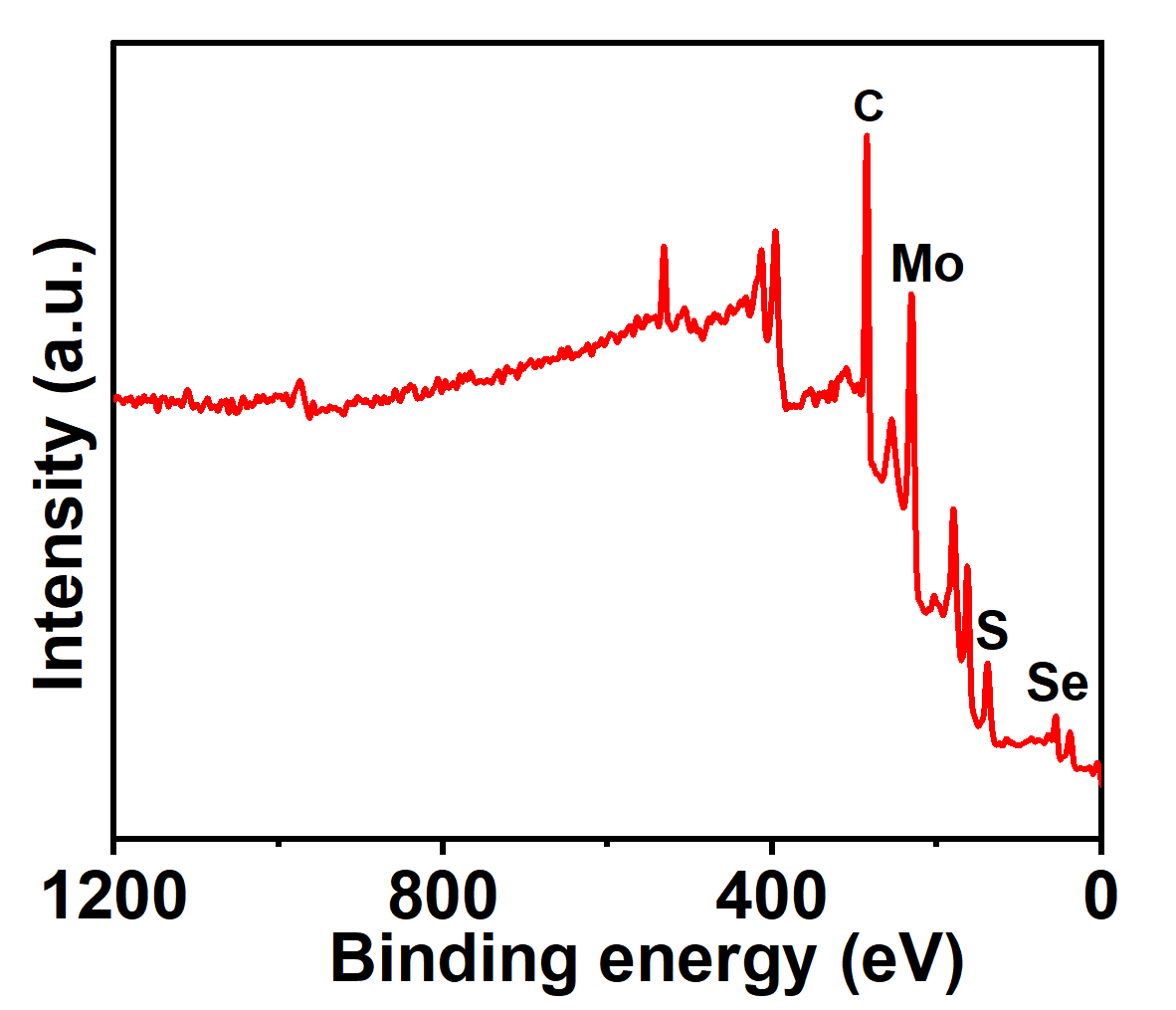


**Fig. S4** XPS of MoSSe@NC composite


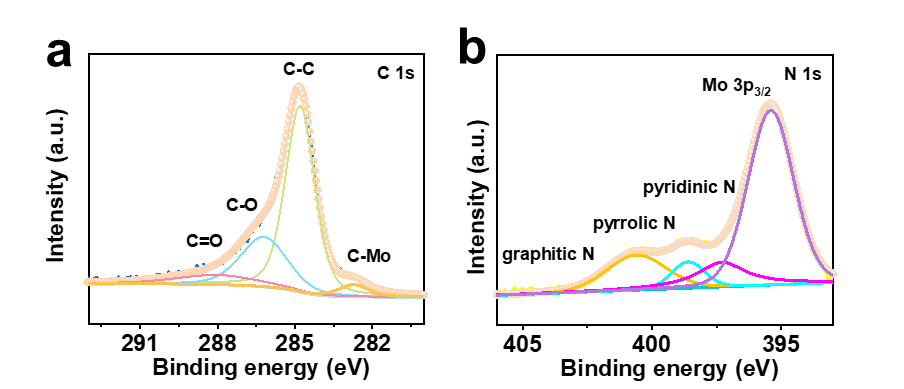


**Fig. S5** XPS spectrum of a) C1s and b) N1s in the MoSSe@NC composite

**Fig. S6** Raman spectra of CNT and MoSSe@NC


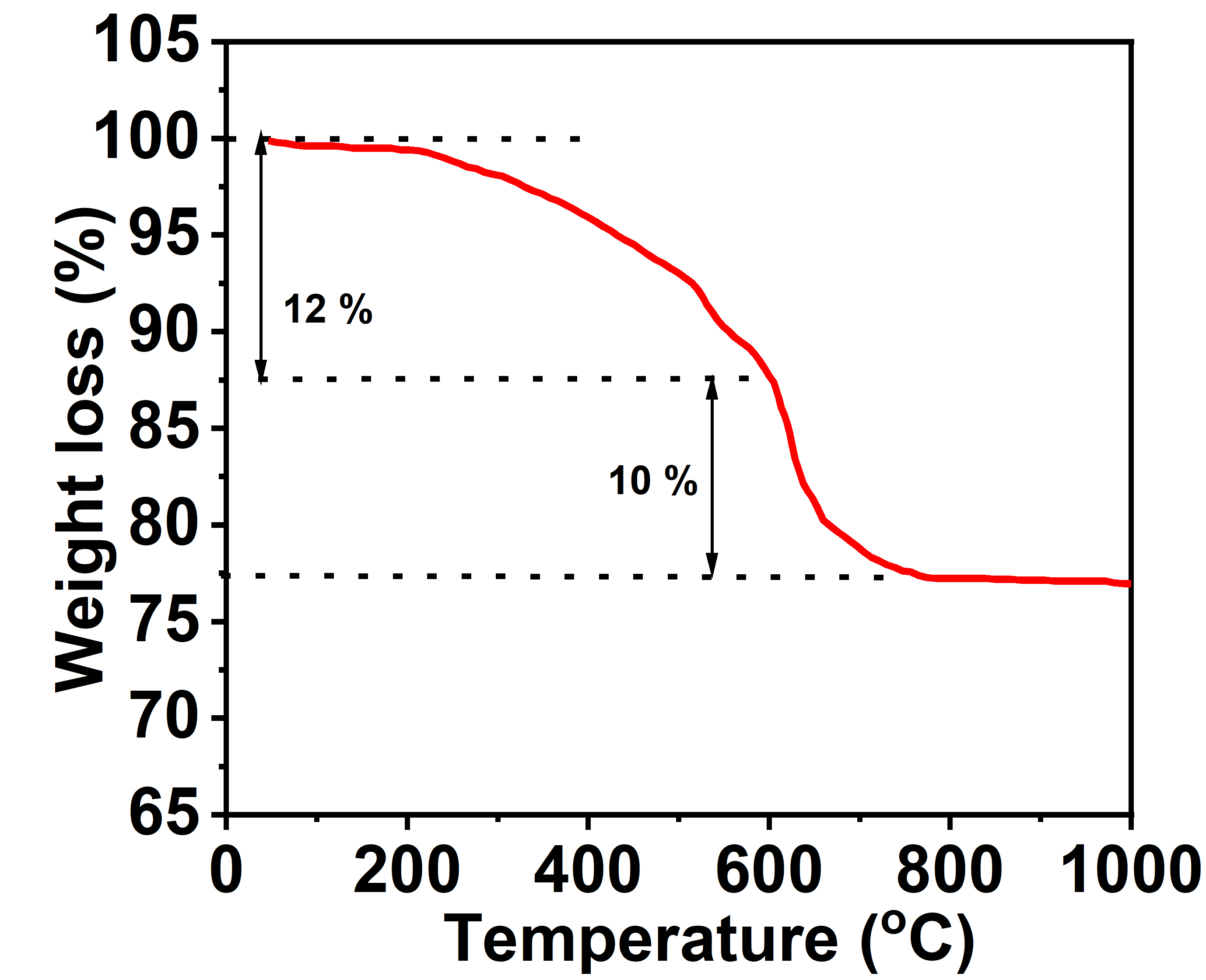


**Fig. S7** TGA profiles of MoSSe@NC

**Fig. S8** N₂ adsorption–desorption isotherm

**Table S1** Comparison of initial Coulombic efficiency with MoS_2_-based anode materials

| **Materials** | **Charge/discharge capacity**  (mAh g^-1^) | **Current density** (mA g^-1^) | **ICE** | **References** |
| --- | --- | --- | --- | --- |
| MoSSe@NC | 466 / 576 | 200 | 70% | Our work |
| the C-p-MoS_2_/C | 666 / 963 | 50 | 69% | Adv. Funct. Mater. 2023, 33, 2207 |
| 1T’’’MoS_2_ | 130 / 240 | 40 | 49% | Adv. Funct. Mater. 2023, 33, 23065 |
| DWHNS Sn/MoS_2_@C, | 445 / 903 | 100 | 49.2% | ACS Nano 2021, 15, 14125−1413 |
| the MoS_2_-on-NC | 304 / 760 | 200 | 40% | Adv. Mater. 2020, 32, 20009 |
| TiNb_2_O_6_@MoS_2_/C | 592 / 1180 | 100 | 51% | J. Mater. Chem. A, 2019, 7, 5760–5768 |

**Table S2** Comparison of the electrochemical performance of the MoSSe@NC electrode with state-of-the-art MoS₂-based anodes reported in the literature

| Materials | Rate/Capcity  (A g^-1^/mAh g^-1^) | Cycling performance | References |
| --- | --- | --- | --- |
| MoSSe@NC | 10 / 176 | 1000 cycles at 3A g^-1^  92% capacity retention | Our work |
| 1T/2H-MoS_2_/NCNHP | 2 / 302 | 500 cycles at 1A g^-1^  70% capacity retention | Small 2020,16, 2004178 |
| 1T’’’ MoS2 | 1 / 75 | 1400 cycles at 1A g^-1^  95% capacity retention | Adv. Funct. Mater.2023, 33,2306550 |
| MoSSe | 5 / 275 | 1000 cycles at 2A g^-1^  75% capacity retention | ACS Nano 2019, 13, 11843-11852 |
| Fe_9_S_10_@MoS_2_@C | 5 / 95 | 50 cycles at 2A g^-1^  80% capacity retention | Energy Storage Materials 24 (2020) 208–219 |
| MoS1.5Se0.5-NC | 5 / 210 | 490 cycles at 5A g^-1^  70% capacity retention | Adv. Energy Mater. 2020, 10, 1904162 |
| CTMG | 5 / 186 | 380 cycles at1A g^-1^  65% capacity retention | Adv. Funct. Mater*.* 2020, *30*, 2001484 |
| MoS_2_/C | 1 / 175 | 300 cycles at1A g^-1^  55% capacity retention | J. Mater. Chem. A, 2019, 7, 5760–5768 |


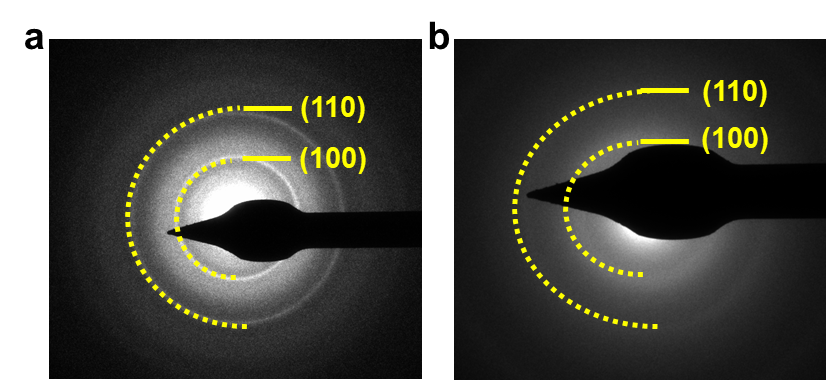


**Fig. S9** SAED patterns of MoSSe@NC: (**a**) before cycling and (**b**) after 500 cycles


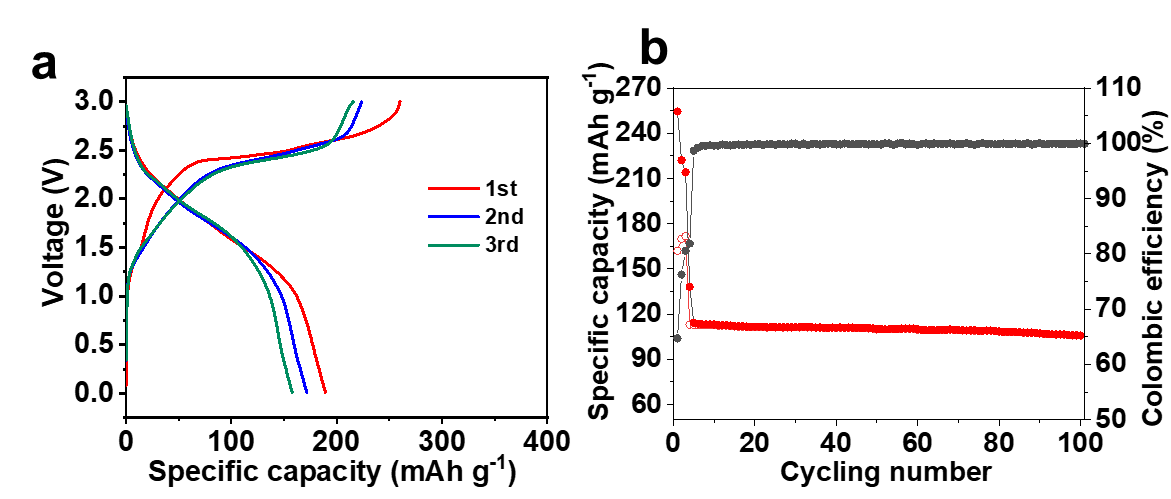


**Fig. S10** (**a**) The charge-discharge profiles and (**b**) Cycling performance at 0.5 A g^-1^ of full cell

The diffusion coefficient can be expressed as the following equation:

$D_{K+}=\frac{4}{\pi}\left( \frac{m_{B}V_{M}}{M_{B}A} \right)^{2}\left( \frac{\Delta E_{s}}{\tau\left( dE_{\tau} \right)/d(\sqrt{\tau})} \right)^{2}$ ($\tau\ll L^{2}/D_{K+}$) (S1)

In this equation, τ(s) is the constant current flux time, m_B_ (g) is the active mass of the electrode, V_M_ (cm^3^ mol^−1^) is the molar volume of the electrode, M_B_ (g mol^-1^) is the molecular weight, A (cm^2^) is the surface area of the electrode, L (cm) is the thickness of the electrode, E_S_ (V) is the total change in cell voltage during a single step and Eτ (V) is the voltage change in the steady state during a single step.

If E versus $\sqrt{\tau}$ shows a linear behavior during the current pulse, the equation can be transformed into:

$D_{K+}=\frac{4}{\pi}\left( \frac{m_{B}V_{M}}{M_{B}A} \right)^{2}\left( \frac{\Delta E_{s}}{\Delta E_{t}} \right)^{2}$ ($\tau\ll L^{2}/D_{K+}$) (S2)


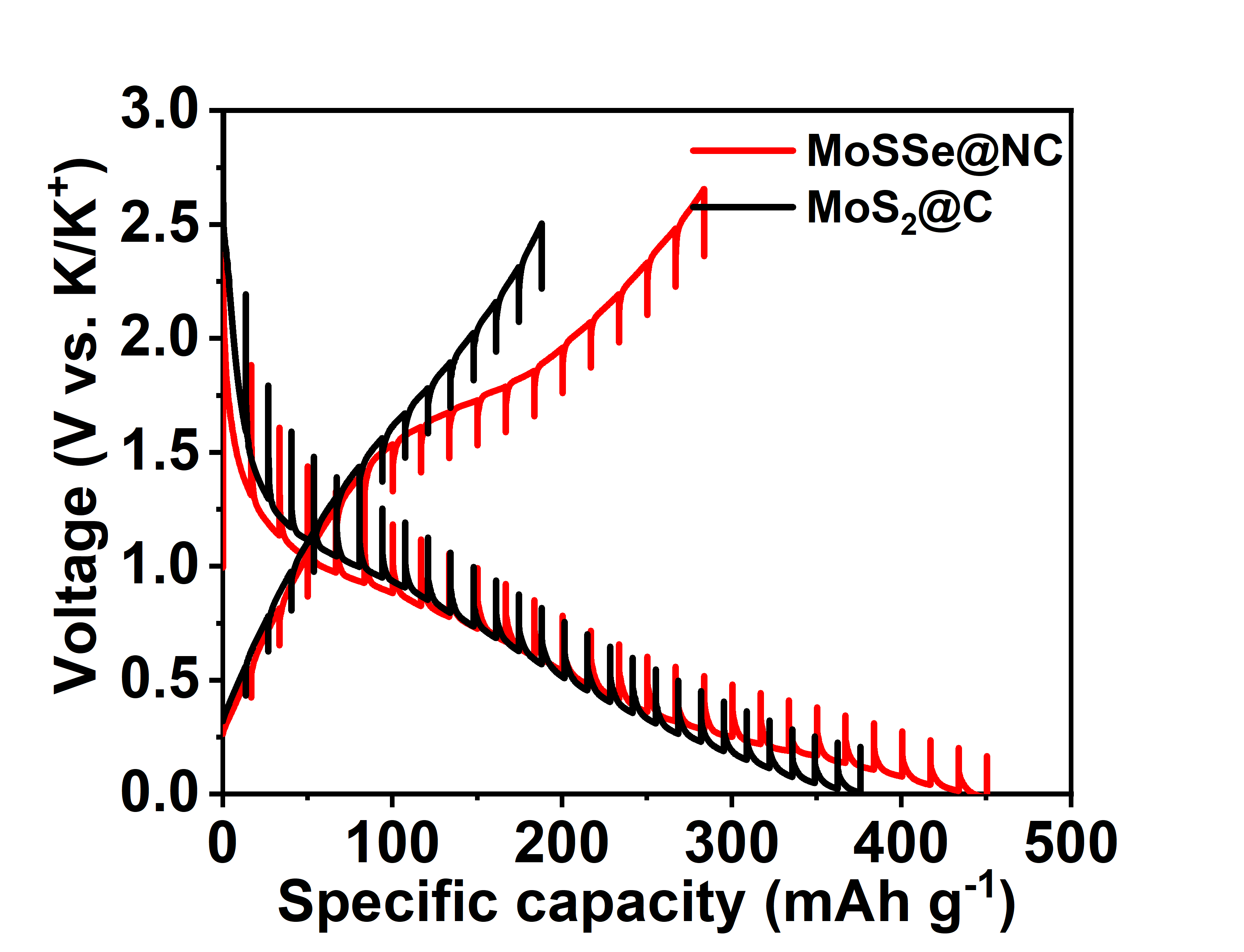


**Fig. S11** GITT profiles of MoSSe@NC and MoS_2_@NC composite

**Fig. S12** EIS spectra of MoS₂@NC and MoSSe@NC

**Fig. S13** The capacitive contribution in the total charge storage at 0.2 mV s^-1^

**Computational Method**

Calculations were conducted by the Gaussian and the Vienna Ab initio Simulation Package (VASP). Projector-augmented-wave (PAW) potentials were used to consider the electron-ion interactions, whereas the electron exchange-correlation interactions were treated using the Perdew-Burke-Ernzerhof (PBE) exchange correlation functional in the scheme of generalized gradient approximation (GGA). For the substrate, a vacuum space of 15 Å placed between adjacent layers to avoid periodic interactions. All the atomic positions and lattice vectors fully optimized using a conjugate gradient algorithm to obtain the unstrained configuration. The Brillouin zone integration employed a gamma-centred 3 × 3 × 1 Monkhorst-Pack mesh of k points. The electron wavefunctions were expanded in a plane wave basis with kinetic energy cutoff of 500 eV. Atomic relaxation performed until the change in total energy was less than 1×10^-5^ eV, and maximum forces were smaller than 0.02 eV/Å.


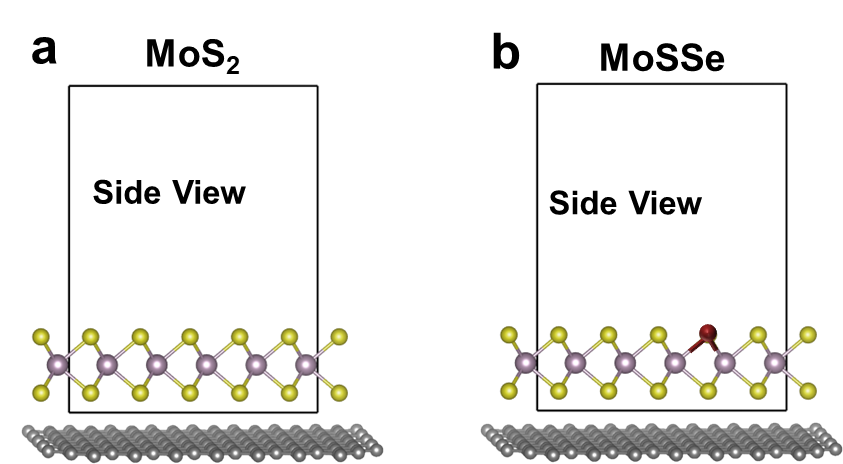


**Fig. S14** the Strucutre of MoS_2_@NC and MoSSe@NC


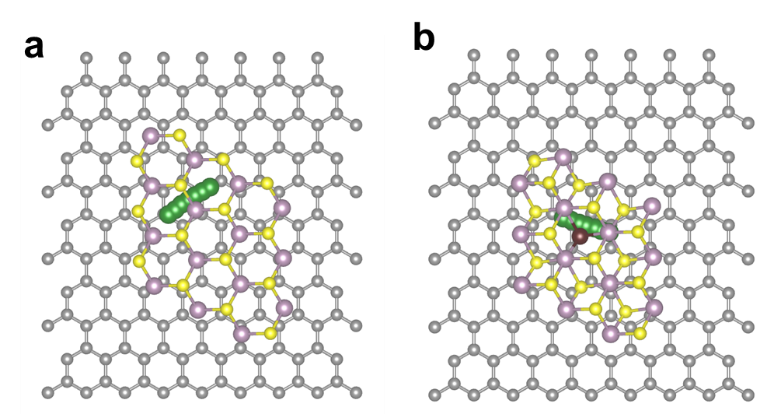


**Fig. S15** The structural diffusion paths of K on MoSSe@NC and MoS₂@NC
